# Persistent hiccups as main COVID-19 manifestation with transient cytotoxic lesion of the corpus callosum splenium during the Omicron wave in the post-vaccination era

**DOI:** 10.1007/s00415-022-11487-z

**Published:** 2022-11-18

**Authors:** Celeste Sassi, Emel Mehmed, Amir Alkhatib, Mario Alberto Forero-Padilla, Dragan S. Goranov, Sylvia Habermann, Sven Rekow, Albert Grüger, Hans-Michael Schmitt

**Affiliations:** 1 Department of Experimental Neurology, Center for Stroke Research Berlin (CSB), Charité-Universitätsmedizin Berlin, Corporate Member of Freie Universität Berlin, Humboldt-Universität Zu Berlin, Berlin Institute of Health, Charitéplatz 1, 10117 Berlin, Germany; 2 Department of Neurology, Martin Gropius Hospital, Eberswalde, Germany

Dear Sirs,

In 2022, the COVID-19 vaccination and the turbulent spreading of the Omicron variant profoundly reshaped the classical COVID-19 alpha and delta phenotype, originally characterized by different degrees of pneumonia associated to a variable neurological spectrum [1].

Due to the lack of case reports these novel post-COVID-19 vaccination and COVID-19 Omicron-associated endophenotypes are to date mostly unexplored, frequently mis- and underdiagnosed and COVID-19 Omicron-associated syndrome remains a diagnosis of exclusion. Here, we describe the first case of a COVID-19 vaccinated patient affected by COVID-19 Omicron infection presenting with isolated persistent hiccups associated to cytotoxic lesion of the corpus callosum (CLOCCs).

A 61-year-old patient was admitted for persistent hiccups occurring ca. 20 times per minute since 4 days. He returned from a vacation in Ireland with very subtle infection symptoms (general weakness, mild headaches and sore throat) 5 days before admission and was tested positive on a PCR test for SARS-CoV-2. He was fully vaccinated and had received the third dose of the Pfizer-*BioNTech* COVID-19 vaccine (BNT162b2) 8 months earlier.

Importantly, he presented both genetic (heterozygous Factor-V-Leiden mutation and positive familial history for deep vein thrombosis) and acquired cardiovascular risk factors (high blood pressure, diabetes mellitus and hyperlipidemia, which were treated with atenolol, metformin and empagliflozin and atorvastatin, respectively). Due to multiple episodes of deep vein thrombosis (superficial femoral vein, V. poplitea and V. tibialis anterior left), in 2012 an oral anticoagulation with phenprocoumon was begun, which in 2017 was switched to rivaroxaban (20 mg once a day). Moreover, due to recurrent depressive episodes he was taking venlafaxine and his past medical history included obstructive sleep apnea and mild hypothyroidism, which was treated with levothyroxine, alcohol abuse leading to liver Cirrhosis (CHILD A) and has been abstinent since 2015.

Beside hiccups he did not present any additional neurological sign and there was no evidence for pneumonia (we did not detect scattered crackles during the auscultation of the lungs and no signs of respiratory insufficiency) or upper respiratory symptoms such as dysgeusia and anosmia. Body temperature was 37 °C and oxygen saturation on room air was 100%. Laboratory tests results displayed typical COVID-19 biomarkers: mild increased C-reactive protein level [18.9 mg/l; normal value (NV): < 5 mg/l], lymphopenia (1000/μl; NV: 1200–3000/μl), thrombocytopenia (110 × 103/μl; NV: 150–362 × 103/μl). Three reverse real-time PCR (rRT-PCR) assays for SARS-CoV-2 on nasopharyngeal swabs were positive and on admission he displayed a very high viral load (rRT-PCR, cycle threshold [CT-value] = 17). The CT-Scan showed a left old thalamic infarct (Fig. 1A). Thin-section chest computed tomography did not display cluster-like ground glass opacities, suggestive of COVID-19 Omicron pneumonia [2] (Fig. 1 B–C). CT-Angiography and ultrasound of the extra and intracranial arteries of the brain did not present vessel stenosis or occlusion, nor specifically any features suggestive of vasculitis (Fig. 1D–K). Brain magnetic resonance imaging (MRI) 1 day after admission confirmed the old left thalamic stroke and revealed a round midline lesion in the splenium of the corpus callosum with subtle hyperintensity on fluid attenuated inversion recovery (FLAIR) (Fig. 1, L) corresponding to a T2-weighted imaging with restricted diffusion (of ca. 5 × 12 mm)(Fig. 1, M), a low signal intensity on apparent diffusion coefficient (ADC) (Fig. 1, N) and a very mild decreased intensity on T1-weighted sequences (Fig. 1, O), compatible with the diagnosis of cytotoxic lesions of the corpus callosum (CLOCCs) [3].

A lumbar puncture was performed after the MRI scans to exclude a possible viral, bacterial or autoimmune encephalitis. Cerebro-spinal fluid (CSF) was crystal clear, with 1/μl white blood cell and normal protein and glucose levels, search for Lyme disease and syphilis tested negative and oligoclonal bands were not present. Given the absence of CSF pleocytosis further screening for neurotropic viruses (Herpes Simplex Virus 1 and 2, Varicella Zoster Virus, Epstein–Barr Virus and cytomegalovirus) was not carried out. Laboratory tests excluded possible endocrine and metabolic disorders (hypoglycemia, hypo- and hyperthyroidism, hypo- and hyperparathyroidism, hypernatremia, renal failure, and hepatic encephalopathy) and iatrogenic causes of CLOCCs were ruled out. Moreover, additional possible infectious diseases such as Herpes zoster were not likely in the absence of the typical painful, blistering skin rash in the trunk along a thoracic dermatome or in the forehead, eyelid and tip of the nose (Herpes zoster ophthalmicus*),* otalgia, auricular vesicles, and peripheral facial paralysis (Herpes zoster oticus).

Finally, calibre changes of the anterior and posterior pericallosal arteries causing a potential ischemic stroke of the splenium of the corpus callosum have not been detected (Fig. 1, J, K).

Due to the very mild symptoms and the ineffectiveness of several monoclonal antibodies such as Sotrovimab against Omicron COVID-19 variants [4] no specific antiviral treatment was given.

After administration of baclofen (10 mg 3 times a day) his hiccups progressively resolved.

Control MRI was performed 17 days later (24 days post- and without residual indication of COVID-19 Omicron infection) and given the complete resolution of the lesion in the splenium of the corpus callosum (Fig. 1, P-S) an additional MRI scan with Gadolinium was not carried out.

CLOCCs is a rare, non-specific and reversible finding on brain MRI, recently described by Tada and colleagues in 2004 [5] that can be caused by a wide range of factors such as medications (typically antiepileptics: carbamazepine, lamotrigine, and phenytoin), infections (viruses: Epstein–Barr, hantavirus, adenovirus, influenza) CNS malignancies, subarachnoid hemorrhage, metabolic disorders (acute renal failure, hypernatremia, hypoglycemia, and hepatic encephalopathy) and migraine with aura.

The splenium of the corpus callosum is particularly vulnerable due to its higher density of glutamate and cytokine receptors. The lesions usually heal within a month of the onset of symptoms [3].

In the past 2 years, a growing body of literature reported CLOCCs associated to COVID-19 alpha and delta variant presenting with a wide clinical spectrum ranging from rapidly developed and fatal systemic inflammatory response syndrome (SIRS) [6] to asymptomatic patients [7] and characterized in most of the reported cases by a mild encephalopathy with reversible splenial lesion (MERS) [8] and to a lesser extent by a severe encephalopathy with reversible splenial lesion (SERS) [9].

To date CLOCCs, due to its rarity and to the lack of repeated MRI scans, is likely to remain underdiagnosed and particularly in the COVID-19 era has been frequently mistaken for an ischemic lesion of the splenium of the corpus callosum[10, 11].

Importantly, despite a significantly higher transmissibility due to an evolutionary very strategic combination of more than 50 mutations, COVID-19-Omicron infection in the post-COVID-19 vaccination era has been described as milder and accompanied by a modest respiratory involvement compared to COVID-19 alpha and delta variant syndrome [12].

However, COVID-19 Omicron-associated clinical features have not been extensively characterized.

We report the first case of COVID-19 Omicron illness post COVID-19 vaccination associated to CLOCCs and persistent hiccups in the absence of any additional neurological or respiratory sign. Importantly, hiccups has been reported in patients carrying COVID-19 alpha and delta variant mostly displaying a severe pneumonia and a peculiar endophenotype has been identified among these patients: particularly affected were male patients, with an average age between 50 and 60 years, with a mild to very severe COVID-19 pneumonia, non-smokers and without any additional clinical sign. Remarkably, due to the prevalent respiratory disease, MRI was not performed in these patients, possibly leading to CLOCCs missed diagnoses [13].

In conclusion, based on our case-report, we suggest that both CLOCCs and hiccups may be distinctive entities and not patho-physiologically linked to pneumonia, as previously hypothesized [6, 13]. On the other hand, CLOCCs and hiccups may be pathogenically correlated rather than being incidental findings as already reported in a patient with neuromyelitis optica [14]. It is plausible that both COVID-19 infection and neuromyelitis optica may be characterized by an acute inflammatory damage, driven by COVID-19 axonal propagation and by an autoimmune process, respectively, synergically affecting both a large bundle of myelinated nerve fibers such as the splenium of the corpus callosum and leading to acute mononeuritis of the phrenic nerve and, consequentially, to a persistent hiccups [15].

Alternatively, given the extensive clinical variability linked to COVID-19-associated CLOCCs, the cytotoxic lesion of the splenium of the corpus callosum, already detectable at the very early stages of COVID-19 infection may represent an unspecific COVID-19 neuroradiological biomarker, likely mirroring the viral load kinetics with maximal hyperintensity on MRI FLAIR and a T2-weighted imaging restricted diffusion corresponding to the highest peak COVID-19 load and progressively decreasing in concert with the viral load slow decline.

Our case-report should foster a validation in large cohorts of COVID-19-Omicron patients.

**Fig. 1:**
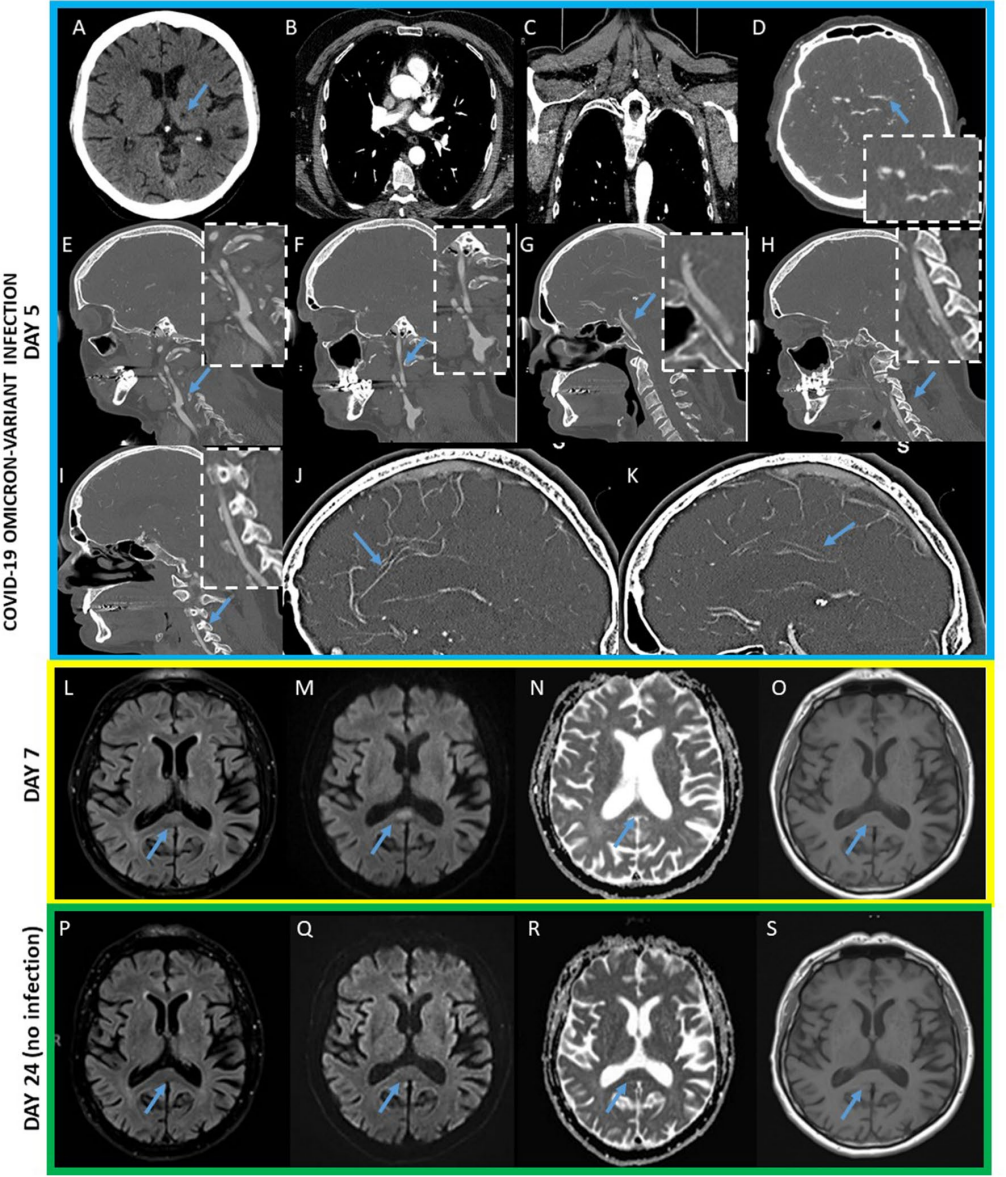
**A-S** Neuroradiological features detected in the patient described. **A–K** CT-Angiography of thorax, head and neck, 5 days post COVID-19 Omicron infection. **A** Axial-CT-Scan displaying an old left thalamic infarct (blue arrow). **B-C** Thorax-CT axial and coronal scans, without suggestive features for a COVID-19 Omicron pneumonia. **D-K** CT-Angiography of head and neck without vessel stenosis or occlusion and nor neuroradiological hallmarks typical for a vasculitis (blue arrows). **D** Circle of Willis (blue arrow). **E** Left extracranial carotid artery (blue arrow). **F** right extracranial carotid artery (blue arrow). **G** Arteria basilaris (blue arrow). **H** Left arteria vertebralis (blue arrow). **I** Right arteria vertebralis (blue arrow). **J** Anterior pericallosal artery (blue arrow). **K** Posterior pericallosal artery (blue arrow). **L–O** axial MRI scans performed 7 days post COVID-19 Omicron infection displaying a round midline lesion in the splenium of the corpus callosum with subtle hyperintensity on fluid attenuated inversion recovery (FLAIR) (**L**) corresponding to a T2-weighted imaging with restricted diffusion (of ca. 5 x 12 mm) (**M**) and low signal intensity on apparent diffusion coefficient (ADC) (**N**) and a very mild hypointense signal on T1-weighted imaging (**O**) (blue arrows). **P**–**S**, axial control MRI scans performed 24 days post COVID-19 Omicron infection presenting a complete resolution of the lesion in the splenium of the corpus callosum in the T2-FLAIR sequences (**P**) as well as in the T2- Diffusion*-*weighted imaging (DWI) (**Q**), on apparent diffusion coefficient (ADC) sequences (**R**) and on T1-weigted imaging (**S**) (blue arrows), patient asymptomatic and SARS-CoV-2 Rapid Antigen Test negative

## Data Availability

The datasets analysed during the current study are available from the corresponding author on reasonable request. Authors have full access to all of the data.
